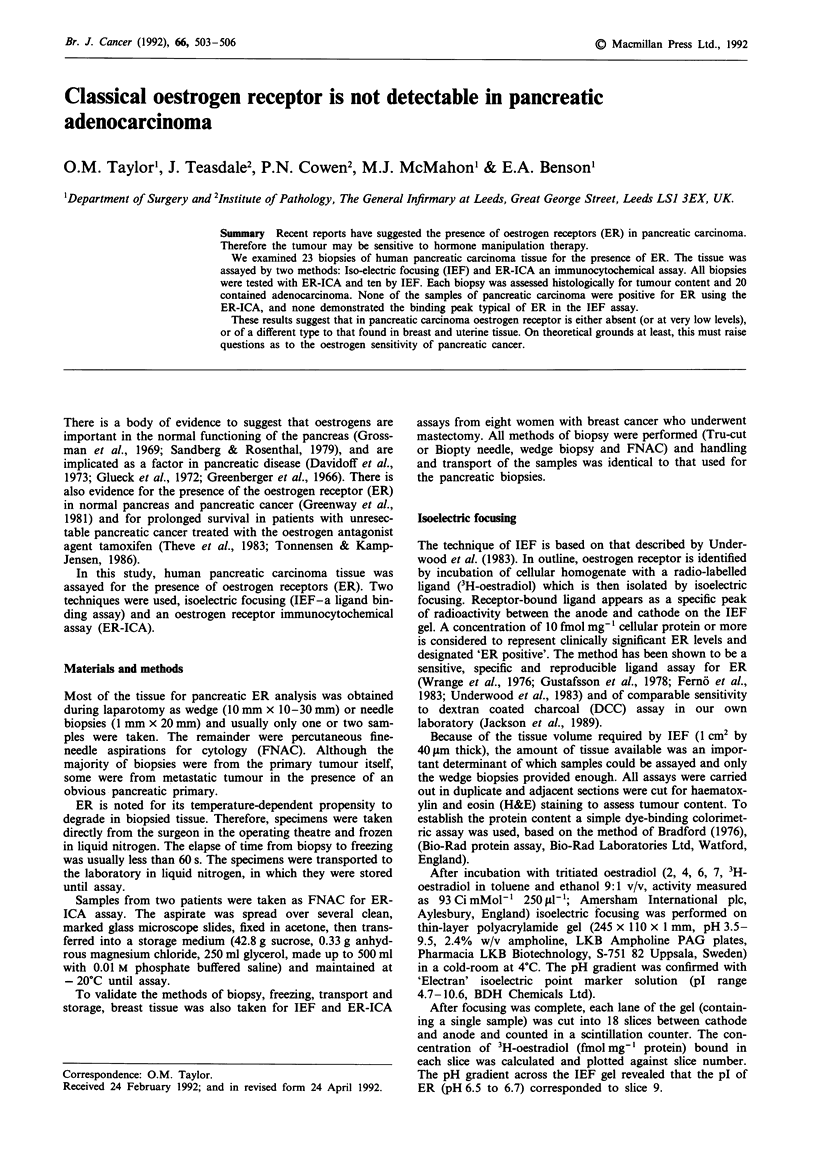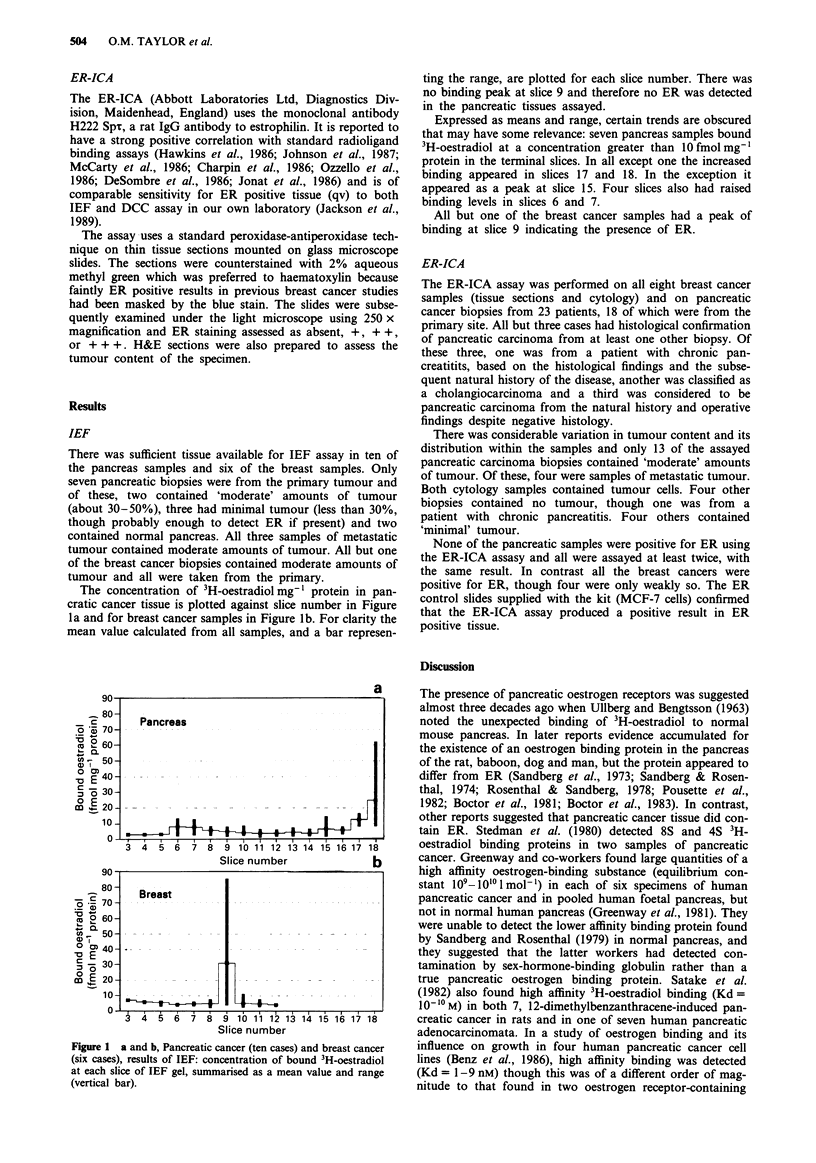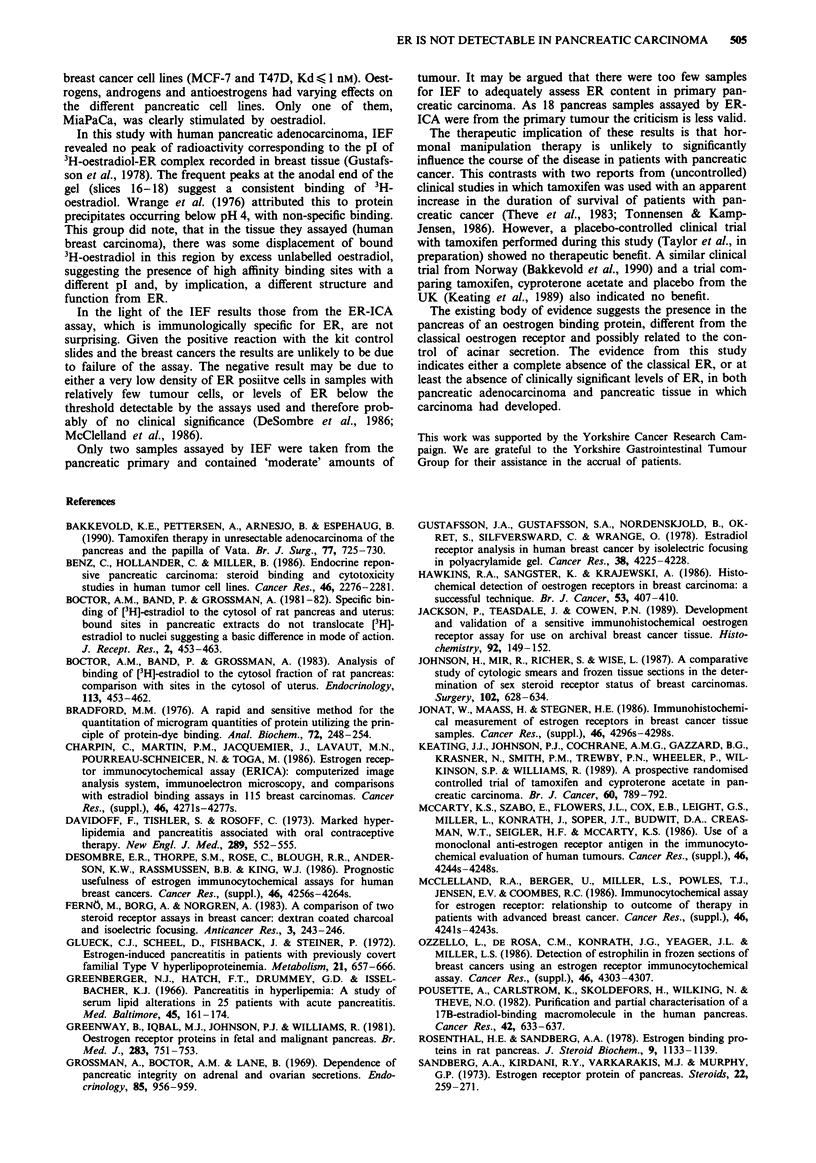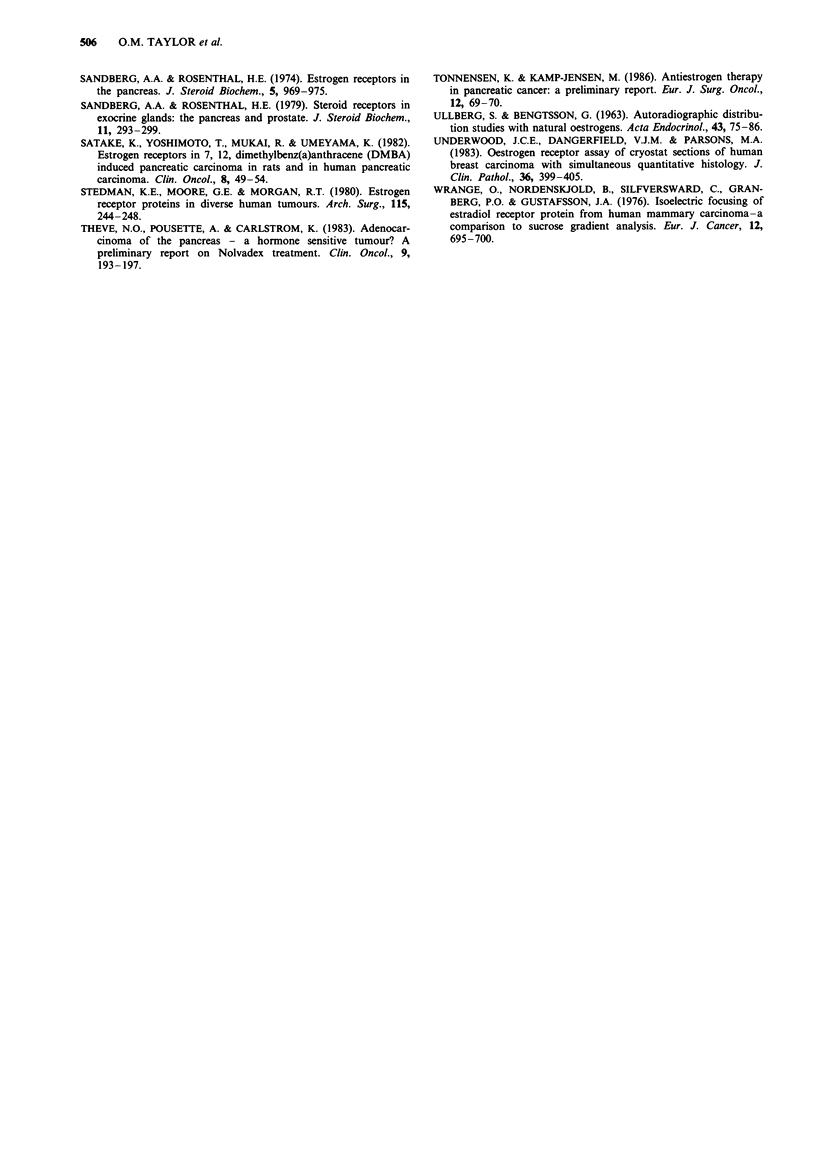# Classical oestrogen receptor is not detectable in pancreatic adenocarcinoma.

**DOI:** 10.1038/bjc.1992.303

**Published:** 1992-09

**Authors:** O. M. Taylor, J. Teasdale, P. N. Cowen, M. J. McMahon, E. A. Benson

**Affiliations:** Department of Surgery, General Infirmary, Leeds, UK.

## Abstract

Recent reports have suggested the presence of oestrogen receptors (ER) in pancreatic carcinoma. Therefore the tumour may be sensitive to hormone manipulation therapy. We examined 23 biopsies of human pancreatic carcinoma tissue for the presence of ER. The tissue was assayed by two methods: Iso-electric focusing (IEF) and ER-ICA an immunocytochemical assay. All biopsies were tested with ER-ICA and ten by IEF. Each biopsy was assessed histologically for tumour content and 20 contained adenocarcinoma. None of the samples of pancreatic carcinoma were positive for ER using the ER-ICA, and none demonstrated the binding peak typical of ER in the IEF assay. These results suggest that in pancreatic carcinoma oestrogen receptor is either absent (or at very low levels), or of a different type to that found in breast and uterine tissue. On theoretical grounds at least, this must raise questions as to the oestrogen sensitivity of pancreatic cancer.


Br. J. Cancer (1992), 66, 503-506 © Macmillan Press Ltd., 1992

### Classical oestrogen receptor is not detectable in pancreatic adenocarcinoma

O.M. Taylor[1], J. Teasdale[2], P.N. Cowen[2], M.J. McMahon[1] & E.A. Benson[1]

[1]*Department of Surgery and* [2]*Institute of Pathology, The General Infirmary at Leeds, Great George Street, Leeds LS1 3EX, UK.*

**Summary** Recent reports have suggested the presence of oestrogen receptors (ER) in pancreatic carcinoma. Therefore the tumour may be sensitive to hormone manipulation therapy.

We examined 23 biopsies of human pancreatic carcinoma tissue for the presence of ER. The tissue was assayed by two methods: Iso-electric focusing (IEF) and ER-ICA an immunocytochemical assay. All biopsies were tested with ER-ICA and ten by IEF. Each biopsy was assessed histologically for tumour content and 20 contained adenocarcinoma. None of the samples of pancreatic carcinoma were positive for ER using the ER-ICA, and none demonstrated the binding peak typical of ER in the IEF assay.

These results suggest that in pancreatic carcinoma oestrogen receptor is either absent (or at very low levels), or of a different type to that found in breast and uterine tissue. On theoretical grounds at least, this must raise questions as to the oestrogen sensitivity of pancreatic cancer.

There is a body of evidence to suggest that oestrogens are important in the normal functioning of the pancreas (Grossman *et al.*, 1969; Sandberg & Rosenthal, 1979), and are implicated as a factor in pancreatic disease (Davidoff *et al.*, 1973; Glueck *et al.*, 1972; Greenberger *et al.*, 1966). There is also evidence for the presence of the oestrogen receptor (ER) in normal pancreas and pancreatic cancer (Greenway *et al.*, 1981) and for prolonged survival in patients with unresectable pancreatic cancer treated with the oestrogen antagonist agent tamoxifen (Theve *et al.*, 1983; Tonnensen & Kamp-Jensen, 1986).

In this study, human pancreatic carcinoma tissue was assayed for the presence of oestrogen receptors (ER). Two techniques were used, isoelectric focusing (IEF-a ligand binding assay) and an oestrogen receptor immunocytochemical assay (ER-ICA).

### Materials and methods

Most of the tissue for pancreatic ER analysis was obtained during laparotomy as wedge (10 mm × 10-30 mm) or needle biopsies (1 mm × 20 mm) and usually only one or two samples were taken. The remainder were percutaneous fine-needle aspirations for cytology (FNAC). Although the majority of biopsies were from the primary tumour itself, some were from metastatic tumour in the presence of an obvious pancreatic primary.

ER is noted for its temperature-dependent propensity to degrade in biopsied tissue. Therefore, specimens were taken directly from the surgeon in the operating theatre and frozen in liquid nitrogen. The elapse of time from biopsy to freezing was usually less than 60 s. The specimens were transported to the laboratory in liquid nitrogen, in which they were stored until assay.

Samples from two patients were taken as FNAC for ER-ICA assay. The aspirate was spread over several clean, marked glass microscope slides, fixed in acetone, then transferred into a storage medium (42.8 g sucrose, 0.33 g anhydrous magnesium chloride, 250 ml glycerol, made up to 500 ml with 0.01 M phosphate buffered saline) and maintained at − 20°C until assay.

To validate the methods of biopsy, freezing, transport and storage, breast tissue was also taken for IEF and ER-ICA assays from eight women with breast cancer who underwent mastectomy. All methods of biopsy were performed (Tru-cut or Biopty needle, wedge biopsy and FNAC) and handling and transport of the samples was identical to that used for the pancreatic biopsies.

### Isoelectric focusing

The technique of IEF is based on that described by Underwood *et al.* (1983). In outline, oestrogen receptor is identified by incubation of cellular homogenate with a radio-labelled ligand ($^3$H-oestradiol) which is then isolated by isoelectric focusing. Receptor-bound ligand appears as a specific peak of radioactivity between the anode and cathode on the IEF gel. A concentration of 10 fmol $mg^{-1}$ cellular protein or more is considered to represent clinically significant ER levels and designated 'ER positive'. The method has been shown to be a sensitive, specific and reproducible ligand assay for ER (Wrange *et al.*, 1976; Gustafsson *et al.*, 1978; Fernö *et al.*, 1983; Underwood *et al.*, 1983) and of comparable sensitivity to dextran coated charcoal (DCC) assay in our own laboratory (Jackson *et al.*, 1989).

Because of the tissue volume required by IEF (1 $cm^2$ by 40 μm thick), the amount of tissue available was an important determinant of which samples could be assayed and only the wedge biopsies provided enough. All assays were carried out in duplicate and adjacent sections were cut for haematoxylin and eosin (H&E) staining to assess tumour content. To establish the protein content a simple dye-binding colorimetric assay was used, based on the method of Bradford (1976), (Bio-Rad protein assay, Bio-Rad Laboratories Ltd, Watford, England).

After incubation with tritiated oestradiol (2, 4, 6, 7, $^3$H-oestradiol in toluene and ethanol 9:1 v/v, activity measured as 93 Ci $mMol^{-1}$ 250 μ$l^{-1}$; Amersham International plc, Aylesbury, England) isoelectric focusing was performed on thin-layer polyacrylamide gel (245 × 110 × 1 mm, pH 3.5-9.5, 2.4% w/v ampholine, LKB Ampholine PAG plates, Pharmacia LKB Biotechnology, S-751 82 Uppsala, Sweden) in a cold-room at 4°C. The pH gradient was confirmed with 'Electran' isoelectric point marker solution (pI range 4.7-10.6, BDH Chemicals Ltd).

After focusing was complete, each lane of the gel (containing a single sample) was cut into 18 slices between cathode and anode and counted in a scintillation counter. The concentration of $^3$H-oestradiol (fmol $mg^{-1}$ protein) bound in each slice was calculated and plotted against slice number. The pH gradient across the IEF gel revealed that the pI of ER (pH 6.5 to 6.7) corresponded to slice 9.

Correspondence: O.M. Taylor.
Received 24 February 1992; and in revised form 24 April 1992.

*ER-ICA*

The ER-ICA (Abbott Laboratories Ltd, Diagnostics Division, Maidenhead, England) uses the monoclonal antibody H222 Spτ, a rat IgG antibody to estrophilin. It is reported to have a strong positive correlation with standard radioligand binding assays (Hawkins *et al.*, 1986; Johnson *et al.*, 1987; McCarty *et al.*, 1986; Charpin *et al.*, 1986; Ozzello *et al.*, 1986; DeSombre *et al.*, 1986; Jonat *et al.*, 1986) and is of comparable sensitivity for ER positive tissue (qv) to both IEF and DCC assay in our own laboratory (Jackson *et al.*, 1989).

The assay uses a standard peroxidase-antiperoxidase technique on thin tissue sections mounted on glass microscope slides. The sections were counterstained with 2% aqueous methyl green which was preferred to haematoxylin because faintly ER positive results in previous breast cancer studies had been masked by the blue stain. The slides were subsequently examined under the light microscope using 250 × magnification and ER staining assessed as absent, +, + +, or + + +. H&E sections were also prepared to assess the tumour content of the specimen.

### Results

*IEF*

There was sufficient tissue available for IEF assay in ten of the pancreas samples and six of the breast samples. Only seven pancreatic biopsies were from the primary tumour and of these, two contained 'moderate' amounts of tumour (about 30–50%), three had minimal tumour (less than 30%, though probably enough to detect ER if present) and two contained normal pancreas. All three samples of metastatic tumour contained moderate amounts of tumour. All but one of the breast cancer biopsies contained moderate amounts of tumour and all were taken from the primary.

The concentration of $^{3}$H-oestradiol $mg^{-1}$ protein in pancratic cancer tissue is plotted against slice number in Figure 1a and for breast cancer samples in Figure 1b. For clarity the mean value calculated from all samples, and a bar representing the range, are plotted for each slice number. There was no binding peak at slice 9 and therefore no ER was detected in the pancreatic tissues assayed.

Expressed as means and range, certain trends are obscured that may have some relevance: seven pancreas samples bound $^{3}$H-oestradiol at a concentration greater than 10 fmol $mg^{-1}$ protein in the terminal slices. In all except one the increased binding appeared in slices 17 and 18. In the exception it appeared as a peak at slice 15. Four slices also had raised binding levels in slices 6 and 7.

All but one of the breast cancer samples had a peak of binding at slice 9 indicating the presence of ER.

*ER-ICA*

The ER-ICA assay was performed on all eight breast cancer samples (tissue sections and cytology) and on pancreatic cancer biopsies from 23 patients, 18 of which were from the primary site. All but three cases had histological confirmation of pancreatic carcinoma from at least one other biopsy. Of these three, one was from a patient with chronic pancreatitits, based on the histological findings and the subsequent natural history of the disease, another was classified as a cholangiocarcinoma and a third was considered to be pancreatic carcinoma from the natural history and operative findings despite negative histology.

There was considerable variation in tumour content and its distribution within the samples and only 13 of the assayed pancreatic carcinoma biopsies contained 'moderate' amounts of tumour. Of these, four were samples of metastatic tumour. Both cytology samples contained tumour cells. Four other biopsies contained no tumour, though one was from a patient with chronic pancreatitis. Four others contained 'minimal' tumour.

None of the pancreatic samples were positive for ER using the ER-ICA assay and all were assayed at least twice, with the same result. In contrast all the breast cancers were positive for ER, though four were only weakly so. The ER control slides supplied with the kit (MCF-7 cells) confirmed that the ER-ICA assay produced a positive result in ER positive tissue.

**Figure 1** **a** and **b**, Pancreatic cancer (ten cases) and breast cancer (six cases), results of IEF: concentration of bound $^{3}$H-oestradiol at each slice of IEF gel, summarised as a mean value and range (vertical bar).

### Discussion

The presence of pancreatic oestrogen receptors was suggested almost three decades ago when Ullberg and Bengtsson (1963) noted the unexpected binding of $^{3}$H-oestradiol to normal mouse pancreas. In later reports evidence accumulated for the existence of an oestrogen binding protein in the pancreas of the rat, baboon, dog and man, but the protein appeared to differ from ER (Sandberg *et al.*, 1973; Sandberg & Rosenthal, 1974; Rosenthal & Sandberg, 1978; Pousette *et al.*, 1982; Boctor *et al.*, 1981; Boctor *et al.*, 1983). In contrast, other reports suggested that pancreatic cancer tissue did contain ER. Stedman *et al.* (1980) detected 8S and 4S $^{3}$H-oestradiol binding proteins in two samples of pancreatic cancer. Greenway and co-workers found large quantities of a high affinity oestrogen-binding substance (equilibrium constant $10^{9}$–$10^{10}$ l $mol^{-1}$) in each of six specimens of human pancreatic cancer and in pooled human foetal pancreas, but not in normal human pancreas (Greenway *et al.*, 1981). They were unable to detect the lower affinity binding protein found by Sandberg and Rosenthal (1979) in normal pancreas, and they suggested that the latter workers had detected contamination by sex-hormone-binding globulin rather than a true pancreatic oestrogen binding protein. Satake *et al.* (1982) also found high affinity $^{3}$H-oestradiol binding (Kd = $10^{-10}$ M) in both 7, 12-dimethylbenzanthracene-induced pancreatic cancer in rats and in one of seven human pancreatic adenocarcinomata. In a study of oestrogen binding and its influence on growth in four human pancreatic cancer cell lines (Benz *et al.*, 1986), high affinity binding was detected (Kd = 1–9 nM) though this was of a different order of magnitude to that found in two oestrogen receptor-containing breast cancer cell lines (MCF-7 and T47D, Kd ≤ 1 nM). Oestrogens, androgens and antioestrogens had varying effects on the different pancreatic cell lines. Only one of them, MiaPaCa, was clearly stimulated by oestradiol.

In this study with human pancreatic adenocarcinoma, IEF revealed no peak of radioactivity corresponding to the pI of $^{3}$H-oestradiol-ER complex recorded in breast tissue (Gustafsson *et al.*, 1978). The frequent peaks at the anodal end of the gel (slices 16–18) suggest a consistent binding of $^{3}$H-oestradiol. Wrange *et al.* (1976) attributed this to protein precipitates occurring below pH 4, with non-specific binding. This group did note, that in the tissue they assayed (human breast carcinoma), there was some displacement of bound $^{3}$H-oestradiol in this region by excess unlabelled oestradiol, suggesting the presence of high affinity binding sites with a different pI and, by implication, a different structure and function from ER.

In the light of the IEF results those from the ER-ICA assay, which is immunologically specific for ER, are not surprising. Given the positive reaction with the kit control slides and the breast cancers the results are unlikely to be due to failure of the assay. The negative result may be due to either a very low density of ER posiitve cells in samples with relatively few tumour cells, or levels of ER below the threshold detectable by the assays used and therefore probably of no clinical significance (DeSombre *et al.*, 1986; McClelland *et al.*, 1986).

Only two samples assayed by IEF were taken from the pancreatic primary and contained 'moderate' amounts of tumour. It may be argued that there were too few samples for IEF to adequately assess ER content in primary pancreatic carcinoma. As 18 pancreas samples assayed by ER-ICA were from the primary tumour the criticism is less valid.

The therapeutic implication of these results is that hormonal manipulation therapy is unlikely to significantly influence the course of the disease in patients with pancreatic cancer. This contrasts with two reports from (uncontrolled) clinical studies in which tamoxifen was used with an apparent increase in the duration of survival of patients with pancreatic cancer (Theve *et al.*, 1983; Tonnensen & Kamp-Jensen, 1986). However, a placebo-controlled clinical trial with tamoxifen performed during this study (Taylor *et al.*, in preparation) showed no therapeutic benefit. A similar clinical trial from Norway (Bakkevold *et al.*, 1990) and a trial comparing tamoxifen, cyproterone acetate and placebo from the UK (Keating *et al.*, 1989) also indicated no benefit.

The existing body of evidence suggests the presence in the pancreas of an oestrogen binding protein, different from the classical oestrogen receptor and possibly related to the control of acinar secretion. The evidence from this study indicates either a complete absence of the classical ER, or at least the absence of clinically significant levels of ER, in both pancreatic adenocarcinoma and pancreatic tissue in which carcinoma had developed.

This work was supported by the Yorkshire Cancer Research Campaign. We are grateful to the Yorkshire Gastrointestinal Tumour Group for their assistance in the accrual of patients.

### References

BAKKEVOLD, K.E., PETTERSEN, A., ARNESJO, B. & ESPEHAUG, B. (1990). Tamoxifen therapy in unresectable adenocarcinoma of the pancreas and the papilla of Vata. *Br. J. Surg.*, **77**, 725–730.

BENZ, C., HOLLANDER, C. & MILLER, B. (1986). Endocrine reponsive pancreatic carcinoma: steroid binding and cytotoxicity studies in human tumor cell lines. *Cancer Res.*, **46**, 2276–2281.

BOCTOR, A.M., BAND, P. & GROSSMAN, A. (1981–82). Specific binding of [$^{3}$H]-estradiol to the cytosol of rat pancreas and uterus: bound sites in pancreatic extracts do not translocate [$^{3}$H]-estradiol to nuclei suggesting a basic difference in mode of action. *J. Recept. Res.*, **2**, 453–463.

BOCTOR, A.M., BAND, P. & GROSSMAN, A. (1983). Analysis of binding of [$^{3}$H]-estradiol to the cytosol fraction of rat pancreas: comparison with sites in the cytosol of uterus. *Endocrinology*, **113**, 453–462.

BRADFORD, M.M. (1976). A rapid and sensitive method for the quantitation of microgram quantities of protein utilizing the principle of protein-dye binding. *Anal. Biochem.*, **72**, 248–254.

CHARPIN, C., MARTIN, P.M., JACQUEMIER, J., LAVAUT, M.N., POURREAU-SCHNEICER, N. & TOGA, M. (1986). Estrogen receptor immunocytochemical assay (ERICA): computerized image analysis system, immunoelectron microscopy, and comparisons with estradiol binding assays in 115 breast carcinomas. *Cancer Res.*, (suppl.), **46**, 4271s–4277s.

DAVIDOFF, F., TISHLER, S. & ROSOFF, C. (1973). Marked hyperlipidemia and pancreatitis associated with oral contraceptive therapy. *New Engl. J. Med.*, **289**, 552–555.

DESOMBRE, E.R., THORPE, S.M., ROSE, C., BLOUGH, R.R., ANDERSON, K.W., RASSMUSSEN, B.B. & KING, W.J. (1986). Prognostic usefulness of estrogen immunocytochemical assays for human breast cancers. *Cancer Res.*, (suppl.), **46**, 4256s–4264s.

FERNÖ, M., BORG, A. & NORGREN, A. (1983). A comparison of two steroid receptor assays in breast cancer: dextran coated charcoal and isoelectric focusing. *Anticancer Res.*, **3**, 243–246.

GLUECK, C.J., SCHEEL, D., FISHBACK, J. & STEINER, P. (1972). Estrogen-induced pancreatitis in patients with previously covert familial Type V hyperlipoproteinemia. *Metabolism*, **21**, 657–666.

GREENBERGER, N.J., HATCH, F.T., DRUMMEY, G.D. & ISSELBACHER, K.J. (1966). Pancreatitis in hyperlipemia: A study of serum lipid alterations in 25 patients with acute pancreatitis. *Med. Baltimore*, **45**, 161–174.

GREENWAY, B., IQBAL, M.J., JOHNSON, P.J. & WILLIAMS, R. (1981). Oestrogen receptor proteins in fetal and malignant pancreas. *Br. Med. J.*, **283**, 751–753.

GROSSMAN, A., BOCTOR, A.M. & LANE, B. (1969). Dependence of pancreatic integrity on adrenal and ovarian secretions. *Endocrinology*, **85**, 956–959.

GUSTAFSSON, J.A., GUSTAFSSON, S.A., NORDENSKJOLD, B., OKRET, S., SILFVERSWARD, C. & WRANGE, O. (1978). Estradiol receptor analysis in human breast cancer by isolelectric focusing in polyacrylamide gel. *Cancer Res.*, **38**, 4225–4228.

HAWKINS, R.A., SANGSTER, K. & KRAJEWSKI, A. (1986). Histochemical detection of oestrogen receptors in breast carcinoma: a successful technique. *Br. J. Cancer*, **53**, 407–410.

JACKSON, P., TEASDALE, J. & COWEN, P.N. (1989). Development and validation of a sensitive immunohistochemical oestrogen receptor assay for use on archival breast cancer tissue. *Histochemistry*, **92**, 149–152.

JOHNSON, H., MIR, R., RICHER, S. & WISE, L. (1987). A comparative study of cytologic smears and frozen tissue sections in the determination of sex steroid receptor status of breast carcinomas. *Surgery*, **102**, 628–634.

JONAT, W., MAASS, H. & STEGNER, H.E. (1986). Immunohistochemical measurement of estrogen receptors in breast cancer tissue samples. *Cancer Res.*, (suppl.), **46**, 4296s–4298s.

KEATING, J.J., JOHNSON, P.J., COCHRANE, A.M.G., GAZZARD, B.G., KRASNER, N., SMITH, P.M., TREWBY, P.N., WHEELER, P., WILKINSON, S.P. & WILLIAMS, R. (1989). A prospective randomised controlled trial of tamoxifen and cyproterone acetate in pancreatic carcinoma. *Br. J. Cancer*, **60**, 789–792.

McCARTY, K.S., SZABO, E., FLOWERS, J.L., COX, E.B., LEIGHT, G.S., MILLER, L., KONRATH, J., SOPER, J.T., BUDWIT, D.A., CREASMAN, W.T., SEIGLER, H.F. & McCARTY, K.S. (1986). Use of a monoclonal anti-estrogen receptor antigen in the immunocytochemical evaluation of human tumours. *Cancer Res.*, (suppl.), **46**, 4244s–4248s.

McCLELLAND, R.A., BERGER, U., MILLER, L.S., POWLES, T.J., JENSEN, E.V. & COOMBES, R.C. (1986). Immunocytochemical assay for estrogen receptor: relationship to outcome of therapy in patients with advanced breast cancer. *Cancer Res.*, (suppl.), **46**, 4241s–4243s.

OZZELLO, L., DE ROSA, C.M., KONRATH, J.G., YEAGER, J.L. & MILLER, L.S. (1986). Detection of estrophilin in frozen sections of breast cancers using an estrogen receptor immunocytochemical assay. *Cancer Res.*, (suppl.), **46**, 4303–4307.

POUSETTE, A., CARLSTROM, K., SKOLDEFORS, H., WILKING, N. & THEVE, N.O. (1982). Purification and partial characterisation of a 17B-estradiol-binding macromolecule in the human pancreas. *Cancer Res.*, **42**, 633–637.

ROSENTHAL, H.E. & SANDBERG, A.A. (1978). Estrogen binding proteins in rat pancreas. *J. Steroid Biochem.*, **9**, 1133–1139.

SANDBERG, A.A., KIRDANI, R.Y., VARKARAKIS, M.J. & MURPHY, G.P. (1973). Estrogen receptor protein of pancreas. *Steroids*, **22**, 259–271.

SANDBERG, A.A. & ROSENTHAL, H.E. (1974). Estrogen receptors in the pancreas. *J. Steroid Biochem.*, **5**, 969–975.

SANDBERG, A.A. & ROSENTHAL, H.E. (1979). Steroid receptors in exocrine glands: the pancreas and prostate. *J. Steroid Biochem.*, **11**, 293–299.

SATAKE, K., YOSHIMOTO, T., MUKAI, R. & UMEYAMA, K. (1982). Estrogen receptors in 7, 12, dimethylbenz(a)anthracene (DMBA) induced pancreatic carcinoma in rats and in human pancreatic carcinoma. *Clin. Oncol.*, **8**, 49–54.

STEDMAN, K.E., MOORE, G.E. & MORGAN, R.T. (1980). Estrogen receptor proteins in diverse human tumours. *Arch. Surg.*, **115**, 244–248.

THEVE, N.O., POUSETTE, A. & CARLSTROM, K. (1983). Adenocarcinoma of the pancreas – a hormone sensitive tumour? A preliminary report on Nolvadex treatment. *Clin. Oncol.*, **9**, 193–197.

TONNENSEN, K. & KAMP-JENSEN, M. (1986). Antiestrogen therapy in pancreatic cancer: a preliminary report. *Eur. J. Surg. Oncol.*, **12**, 69–70.

ULLBERG, S. & BENGTSSON, G. (1963). Autoradiographic distribution studies with natural oestrogens. *Acta Endocrinol.*, **43**, 75–86.

UNDERWOOD, J.C.E., DANGERFIELD, V.J.M. & PARSONS, M.A. (1983). Oestrogen receptor assay of cryostat sections of human breast carcinoma with simultaneous quantitative histology. *J. Clin. Pathol.*, **36**, 399–405.

WRANGE, O., NORDENSKJOLD, B., SILFVERSWARD, C., GRANBERG, P.O. & GUSTAFSSON, J.A. (1976). Isoelectric focusing of estradiol receptor protein from human mammary carcinoma – a comparison to sucrose gradient analysis. *Eur. J. Cancer*, **12**, 695–700.